# Gefitinib treatment affects androgen levels in non-small-cell lung cancer patients

**DOI:** 10.1038/sj.bjc.6602585

**Published:** 2005-05-03

**Authors:** M Nishio, F Ohyanagi, A Horiike, Y Ishikawa, Y Satoh, S Okumura, K Nakagawa, K Nishio, T Horai

**Affiliations:** 1 Division of Internal Medicine, Cancer Institute Hospital, Japanese Foundation For Cancer Research, Ariake 3-10-6, Koto-ku, Tokyo 135-8550, Japan; 2 Department of Chest Surgery, Cancer Institute Hospital, Japanese Foundation For Cancer Research, Tokyo, Japan; 3 Department of Pathology, Cancer Institute, Japanese Foundation For Cancer Research, Tokyo, Japan; 4 Pharmacology Division, National Cancer Center Research Institute, Tokyo, Japan

**Keywords:** sex hormone, epidermal growth factor receptor, tyrosine kinase inhibitor

## Abstract

Gefitinib, an inhibitor of the epidermal growth factor receptor (EGFR, HER1/ErbB1) tyrosine kinase, has been shown to have clinical activity against non-small-cell lung cancers (NSCLCs), especially in women nonsmokers with adenocarcinomas. The aim of the present study was to clarify the relationship between androgen levels and gefitinib treatment in patients with advanced NSCLCs. Sera from 67 cases (36 men and 31 women) were obtained pretreatment and during treatment with gefitinib monotherapy (days 14–18) for examination of testosterone, dehydroepiandrosterone sulphate (DHEA), and dehydroepiandrosterone sulphate (DHEAS) levels. Testosterone and DHEA during treatment were significantly lower than the pretreatment values in both women and men, and the DHEAS levels during treatment were also significantly lowered in women. Gefitinib treatment significantly suppressed androgen levels, especially in women who had no smoking history. In addition, hormone levels in women responding to gefitinib were significantly lower during the treatment than in women who did not respond. Gefitinib-associated decrease in serum androgen levels may play a role in its clinical efficacy.

Non-small-cell lung cancer (NSCLC) is a major health problem worldwide for both men and women (Ferlay *et al*, 2001). Usually at the time of diagnosis more than 50% of the patients have advanced or metastatic disease. While cytotoxic chemotherapy slightly prolongs survival among advanced NSCLC patients, it exerts clinically significant adverse effects (Non-Small-Cell Lung Cancer Collaborative Group, 1995; Schiller *et al*, 2002). An effective, palliative, low-toxicity treatment for patients with advanced NSCLC is therefore needed and for this purpose the epidermal growth factor receptor (EGFR/HER1) is a promising target. Gefitinib (ZD 1839, Iressa; AstraZeneca, London, UK) is an orally active, selective HER1-tyrosine kinase inhibitor (Wakeling *et al*, 2002), which has been shown to elicit objective responses in NSCLC cases, particularly in women nonsmokers with adenocarcinomas (Fukuoka *et al*, 2003; Kris *et al*, 2003). Recently, active mutations of EGFR have been identified in such cases (Paez *et al*, 2004; Pao *et al*, 2004) and may be linked with the sensitivity to gefitinib (Lynch *et al*, 2004; Paez *et al*, 2004; Pao *et al*, 2004). However, the reason why mutations frequently occur in these particular individuals is poorly understood.

Androgens are important hormones that play definitive roles in the differentiation of males and females. They can modify the activity of the epidermal growth factor network and EGFR signaling is essential for androgen-induced proliferation (Klein and Nielsen, 1993; Dammann *et al*, 2000; Torring *et al*, 2003). A receptor for androgens has been reported to occur in NSCLCs (Beattie *et al*, 1985; Kaiser *et al*, 1996) and there may be cooperative interaction between the hormones and active mutations of EGFR during the development of lung cancer. Previous reports have suggested that smoking increases the levels of androgens in men and women (Law *et al*, 1997; Trummer *et al*, 2002) and carcinogens from cigarette smoke may disrupt androgen function by reducing androgen receptor (AR) levels in androgen-responsive organs (Lin *et al*, 2004).

On the basis of these reports, we hypothesised that androgens may play an important role in the efficacy of gefitinib in NSCLC cases. In the present study, we therefore evaluated androgen levels in patients treated with gefitinib and the relationship with clinical efficacy.

## PATIENTS AND METHODS

Between September 2002 and May 2004, 67 advanced or recurrent NSCLC patients were analysed in this study. All 67 were treated at our institution with gefitinib monotherapy (250 mg oral doses of gefitinib once daily) until disease progression occurred. Response evaluation and confirmation were performed in accordance with the WHO criteria (WHO, 1979). In brief, complete response (CR) was defined as complete disappearance of all lesions in imaging studies for at least 4 weeks without the appearance of any new lesions. Partial response (PR) was defined as a >50% decrease under the baseline in the sum of the products of the perpendicular diameters of all measurable lesions and at least stabilisation of all nonmeasurable lesions over a minimum period of 4 weeks. Progressive disease (PD) was defined as a >25% increase in the sum of the products of all measurable lesions, an unequivocal increase of nonmeasurable disease, or the appearance of new lesions. Cases were classified as having stable disease (SD) if none of the criteria for classifying responses as a CR, PR, or PD were met.

Blood was drawn before and during gefitinib administration. A previous report indicated the median time to symptom improvement with gefitinib to be only 8 days (Fukuoka *et al*, 2003), and we therefore checked the hormone levels at days 14–18, when serum was sampled between 10:00 and 14:00 and stored at 80°C for subsequent analyses. Serum levels of testosterone, dehydroepiandrosterone (DHEA), and dehydroepiandrosterone sulphate (DHEAS) were all measured at the SRL Laboratory (Tokyo, Japan). For testosterone, an electrochemiluminescence immunoassay was applied (ECLusys testosterone; Roche Diagnostics KK, Tokyo, Japan) and radioimmunoassays were used for DHEA and DHEAS (DPC DHEA and DPC DHEAS kits; Diagnostic Products Corporation, Los Angeles, CA, USA). The detection limits for testosterone, DHEA, and DHEAS were 5, 0.2, and 20 ng ml^−1^, respectively. Inter- and intra-assay coefficients of variation were 6 and 8% for testosterone, 8 and 9% for DHEA, and 4 and 4% for DHEAS, respectively.

Appropriate ethical review boards approved the study, which followed the recommendations of the Declaration of Helsinki for biomedical research involving human subjects.

### Statistical analysis

A paired *t*-test was used to compare the androgen levels between the two time periods. Patients were grouped into responders (CR and PR) and nonresponders (SD and PD) and the variables in each group were compared with an unpaired *t*-test. All statistical analyses were performed using SPSS version 8 statistical software (SPSS Inc., IL, USA).

## RESULTS

### Patient characterisation

Data for patient characteristics are listed in Table 1. Of the 67, 31 (46.3%) were women. The median age was 61 years (range, 42–80 years). There were 26 patients (38.8%) who had never smoked and adenocarcinoma was the primary histological finding in 56 cases (83.6%). There was no prior chemotherapy in 16 (23.9%) of the patients, and the remainder had received platinum-based chemotherapy.

Response to treatment could only be evaluated in 64 of the 67 cases. We observed 20 PR (29.8%), and of these, 13 (65%) were women and seven (35%) were men (*P*=0.074). The median and range of treatment duration with gefitinib were 2.1 and 0.2–21 months. In all, 10 (50%) of 20 responders and 29 (66%) of 44 nonresponders had a smoking history (*P*=0.226).

### Effects of gefitinib treatment on androgens levels in NSCLC patients

Testosterone, DHEA, and DHEAS were detected in the serum of all 67 patients (see Table 2). There was a significant difference observed between men and women for serum testosterone levels (*P*<0.0001), but not for serum DHEA or DHEAS (DHEA; *P*=0.267, DHEAS; *P*=0.0565).

**Figure 1:**
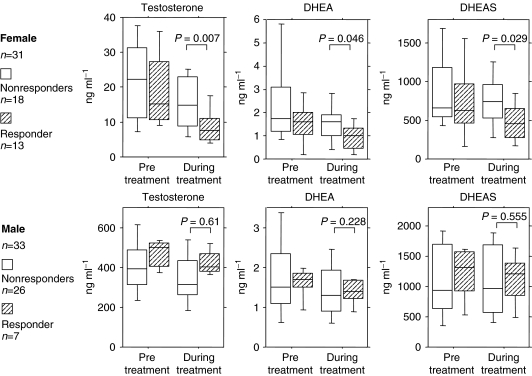
Serum testosterone, DHEA, and DHEAS levels, pretreatment and during the gefitinib administration. Each androgen levels are depicted in accordance to clinical response of gefitinib treatment (responders, PR; nonresponders, SD or PD). Error bars showed standard deviation.

In women, testosterone, DHEA, and DHEAS levels at pretreatment were significantly higher than during treatment (testosterone; *P*=0.025, DHEA; *P*=0.0065, DHEAS; *P*=0.0326). In men, pretreatment testosterone and DHEA levels were significantly higher than during treatment, but there was no significant difference for DHEAS (testosterone, *P*=0.0009; DHEA, *P*=0.0085; DHEAS, *P*=0.33). In addition, we compared hormone levels between smokers and nonsmokers. Pretreatment, there were no significant differences between women with and without a smoking history. On the other hand, hormone levels were significantly suppressed by gefitinib treatment in the 21 women who had no smoking history (testosterone, *P*=0.0016; DHEA, *P*=0.0157; DHEAS, *P*=0.0441), but not in the 10 who had a smoking history (testosterone, *P*=0.6159; DHEA, *P*=0.2487; DHEAS, *P*=0.4740). Figure 1 depicts the androgen levels for women after dividing the group into responders *vs* nonresponders. Testosterone, DHEA, and DHEAS levels in women responders during treatment were significantly lower than those observed in women nonresponders (testosterone, *P*=0.007; DHEA, *P*=0.046; DHEAS, *P*=0.029). When men were included in the analysis, DHEA and DHEAS levels during treatment in the responders (*n*=20) were still significantly lower than in the nonresponders (*n*=44) (DHEA, *P*=0.0324; DHEAS, *P*=0.0447).

## DISCUSSION

The present study of androgen levels (testosterone, DHEA, and DHEAS) in advanced NSCLC patients treated with gefitinib monotherapy revealed treatment-related decrease, especially in women who had no smoking history. The clinical response of gefitinib treatment appeared to be correlated with the suppression of the hormone levels.

To our knowledge, there have been no previous reports of effects of gefitinib treatment on levels of androgens in patients, although a number of authors have examined relationships between androgens and activity of the epidermal growth factor network (Klein and Nielsen, 1993; Dammann *et al*, 2000; Torring *et al*, 2003). There is evidence that EGFR expression is involved in prostate cancer development and in progression to androgen independence (Di Lorenzo *et al*, 2002), and an *in vitro* study has provided evidence that androgens increase the EGFR levels in androgen-sensitive prostate cancer cells and that EGFR signaling is essential for androgen-induced proliferation and survival (Torring *et al*, 2003). Although there has been no indication of any relationship between androgens and EGFR in NSCLCs, expression of ARs has been detected in NSCLC cell lines and biopsy samples of primary lung cancers (Kaiser *et al*, 1996). Additionally, expression has been detected more frequently in women with adenocarcinoma, and thus this may be a prognostic factor for use of gefitinib in NSCLCs (Fukuoka *et al*, 2003; Kris *et al*, 2003; Miller *et al*, 2004). The data suggest that there is a correlation between the AR and EGFR functions in lung cancer. In agreement with this hypothesis, our results demonstrated clinical responses to gefitinib treatment to correlate with suppression of androgen levels.

One reason for lower androgen levels in responders than nonresponders might be that smokers are resistant and have higher androgen levels. However, there were no significant difference in smoking history between responders and nonresponder in our study and there was no significant difference of the pretreatment levels of androgens between smokers and nonsmokers. On the other hand, gefitinib treatment significantly suppressed androgen levels in women who had no smoking history, but not in smokers. Smoking may disrupt the correlation between EGFR and androgen.

Both gefitinib and androgens are metabolised by CYP3A4/5; therefore, it can be speculated that gefitinib may affect the metabolisms of androgens. On the other hand, there are no direct evidences demonstrating PK interaction between gefitinib and androgens. PK interaction between gefitinib and other drugs metabolised by CY3A4/5 such as docetaxel or irinotecan were reported (Fandi *et al*, 2003; Furman *et al*, 2004). These reports suggested that gefitinib may decrease the clearance of these drugs and it may be due to CYP3A4/5 substrate competition. If there are any PK interactions between gefitinib and androgens, androgens clearance may decrease and androgen levels may increase by gefitinib treatment. However, we showed that gefitinib treatment decreased the levels of androgens and it suggested that the effect may not be due to change of CYP3A4/5 activity.

With single estimations of testosterone and DHEA, it is necessary to take into account the circadian rhythms. In this study, all blood was therefore taken at approximately the same time, that is, between 10:00 and 14:00, although this does not preclude any influence of cycles. On the other hand, several reports have suggested that there is no circadian rhythm for serum DHEAS levels (Molta and Schwartz, 1986; Hall *et al*, 1993; Kos-Kudla *et al*, 2001). Therefore, the differences seen in the DHEAS levels in this study presumably reflect actual effects of gefitinib treatment. This would suggest that the data for the other hormones might also have clinical significance.

In conclusion, the results of the present small, retrospective study indicate that androgen levels in NSCLC patients are affected by gefitinib treatment and that they may be factors determining sensitivity to this chemotherapeutic agent. Further large-scale prospective trials are needed in the future to confirm these results and to examine inter-relationships among androgens, smoking, gefitinib sensitivity, and EGFR mutations.

## Figures and Tables

**Table 1:** Patient characteristics

**Variable**	**No. of patients**	**%**
Total	67	
		
*Sex*		
Male	36	53.7
Female	31	46.3
		
*Age (years)*		
Median	61	
Range	42–80	
		
*Smoking history*		
Never	26	38.8
Former/current	41	61.2
		
*Performance status*		
0, 1	48	71.6
>2	19	28.4
		
*Histology*		
Ad	56	83.6
Non-Ad	11	16.4
		
*Stage*		
II–III	17	25.4
IV	27	40.3
Recurrence after surgery	23	34.3
		
*Response*		
PR	20	29.8
SD/PD	44	64.7
NE	3	4.5
		
*Prior chemotherapy*		
No	16	23.9
Yes	51	76.1

Ad=adenocarcinoma; non-Ad=nonadenocarcinoma; PR=partial response; SD=stable disease; PD=progressive disease; NE=not evaluable.

**Table 2:** Androgen levels in patients treated with gefitinib

	**Pretreatment**		**During treatment**		
**Variable**	***n***	**Mean±s.d.**	***n***	**Mean±s.d.**	**Paired *t***-**test**
*Testosterone* (*ng ml*^−*1*^)					
Female		21.5±12.0	31	13.8.0±11.0	*P*=0.025
Male	37	409.7±129.8	37	350.8±135.7	*P*=0.0009
					
*DHEA* (*ng ml*^−*1*^)					
Female	31	2.21±2.03	31	1.33±0.83	*P*=0.0065
Male	37	1.78±1.06	37	1.49±0.92	*P*=0.0085
					
*DHEAS* (*ng ml*^−*1*^)					
Female	31	854.4±579.5	31	645.8±365.6	*P*=0.0326
Male	37	1137.4±607.7	37	1103.0±601.5	*P*=0.33

s.d.=standard deviation; DHEA=dehydroepiandrosterone; DHEAS=dehydroepiandrosterone sulphate.
